# Arnica Poisoning

**Published:** 1883-10-20

**Authors:** Wm. A. Thom

**Affiliations:** Norfolk, Va.


### ARNICA POISONING. DOSE—ONE OUNCE OF THE TINCTURE.

BY WM. A. THOM, JR., M. D., NORFOLK, VA.

On Friday, April 6th, I was called to see Luther Phillips, a negro laborer, aged 24 years, who four hours before had taken a fluid ounce of the tincture of arnica. I found him lying in a state of absolute insensibility, breathing eighteen times to the minute; not stertorous; pulse 100, full and strong; temperature normal; pupils slightly contracted, but not sufficiently so to attract attention without close examination. He was so thoroughly insensible that the application of the flame of a lamp failed to produce the slightest reflex action; the conjunctivæ were without sensation. I

was told that half an hour after taking the arnica he had been found lying as I found him; there was no vomiting or purging. Remembering Dr. Bertin's case, published in the London Lancet of November 19th, 1864, in which collapse came on ten hours after the ingestion of the drug, I at once ordered drachm doses of brandy every fifteen minutes, with hot pediluvia every half hour. At six o'clock p. m., ten hours after taking the arnica, he began to show signs of returning sensibility, only manifested by reflex response to cutaneous irritation. At twelve at night he became wildly delirious, remaining in this state for several hours, after which he fell asleep, awakening on the morning of the second day with a dizziness and inability to walk straight; great burning pain throughout the alimentary tract. During the day he had three very large watery evacuations, with profuse diuresis. On the third day he was quite well.

The chief interest of this case lies in the fact of its extreme rarity. In Bertin's case there were no symptoms until the collapse came on ten hours after taking the drug. In mine the effects were immediate, and there was never the slightest sign of collapse. In both, brandy and hot water were used with like good effects.—*Virginia Med. Monthly.*

